# Asynchronous excitatory neuron development in an isogenic cortical spheroid model of Down syndrome

**DOI:** 10.3389/fnins.2022.932384

**Published:** 2022-09-07

**Authors:** Zhen Li, Jenny A. Klein, Sanjeev Rampam, Ronni Kurzion, Natalie Baker Campbell, Yesha Patel, Tarik F. Haydar, Ella Zeldich

**Affiliations:** ^1^Center for Neuroscience Research, Children’s National Hospital, Washington, DC, United States; ^2^Graduate Program for Neuroscience, Boston University, Boston, MA, United States; ^3^Department of Biomedical Engineering, Boston University, Boston, MA, United States; ^4^Department of Chemistry, Boston University, Boston, MA, United States; ^5^Department of Anatomy and Neurobiology, Boston University, Boston, MA, United States; ^6^Department of Biochemistry and Molecular Biology, University of Massachusetts Amherst, Amherst, MA, United States

**Keywords:** trisomy, cerebral organoids, isogenic iPSCs, scRNA-seq, developmental asynchrony, neuronal motility, brain organoids

## Abstract

The intellectual disability (ID) in Down syndrome (DS) is thought to result from a variety of developmental deficits such as alterations in neural progenitor division, neurogenesis, gliogenesis, cortical architecture, and reduced cortical volume. However, the molecular processes underlying these neurodevelopmental changes are still elusive, preventing an understanding of the mechanistic basis of ID in DS. In this study, we used a pair of isogenic (trisomic and euploid) induced pluripotent stem cell (iPSC) lines to generate cortical spheroids (CS) that model the impact of trisomy 21 on brain development. Cortical spheroids contain neurons, astrocytes, and oligodendrocytes and they are widely used to approximate early neurodevelopment. Using single cell RNA sequencing (scRNA-seq), we uncovered cell type-specific transcriptomic changes in the trisomic CS. In particular, we found that excitatory neuron populations were most affected and that a specific population of cells with a transcriptomic profile resembling layer IV cortical neurons displayed the most profound divergence in developmental trajectory between trisomic and euploid genotypes. We also identified candidate genes potentially driving the developmental asynchrony between trisomic and euploid excitatory neurons. Direct comparison between the current isogenic CS scRNA-seq data and previously published datasets revealed several recurring differentially expressed genes between DS and control samples. Altogether, our study highlights the power and importance of cell type-specific analyses within a defined genetic background, coupled with broader examination of mixed samples, to comprehensively evaluate cellular phenotypes in the context of DS.

## Introduction

Down syndrome (DS) is the most common genetic form of intellectual disability (ID), caused by triplication of human chromosome 21 (HSA21), with a prevalence of one in 700 live births in the United States (Mai et al., 2019). HSA21 contains more than 310 genes, and its triplication causes wide-spread molecular and cellular changes that underlie the characteristic phenotypes associated with DS (Vohr et al., 1989; Olmos-Serrano et al., 2016). The ID in individuals with DS is presumed to arise from anatomical and physiological alterations of the brain during atypical neurodevelopment. Histological abnormalities in brains from individuals with DS are evident as early as late-gestation, including delayed cortical lamination, reduced cerebral volume, hypocellularity, and altered neural processes (Haydar and Reeves, 2012; Ábrahám et al., 2012; Olmos-Serrano et al., 2016). These anatomical changes are, in turn, a product of cellular changes in the embryonic brain, including abnormal divisions of neural progenitors, aberrant neuronal migration, and altered cell-to-cell adhesion (Tyler and Haydar, 2013; Huo et al., 2018; Bells et al., 2019). However, molecular processes underlying these cellular, anatomical and physiological changes that result in ID have not been fully elucidated yet.

On one hand, the lack of mechanistic knowledge is due in part to the limited access to and ethical considerations of conducting research in human brain tissue, which restricts our ability to temporally examine how trisomy affects the development of different types of brain cells. On the other hand, mouse models of DS, while invaluable, are challenged by inconsistency in genetic backgrounds, reduced mutation penetration, and phenotypic drift (Gardiner et al., 2003; Gupta et al., 2016; Kazuki et al., 2020; Shaw et al., 2020). Thus, to model human- and disease-relevant aspects of DS, *in vitro* cultures of human induced pluripotent stem cells (iPSCs) have risen in popularity, due to their ability to reflect regional and cell type-specific features of the human brain. Three dimensional (3D) cortical spheroids (CS) and organoids have been shown to surpass two dimensional (2D) iPSC cultures in recapitulating signaling pathways, patterning, fate acquisition, and developmental trajectories of the *in vivo* environment (Kathuria et al., 2020). CS have also been shown to better preserve the expression of cell adhesion molecules, extracellular matrix components, and cell membrane structures (Scuderi et al., 2021) and possess a greater transcriptomic overlap with human fetal brain at mid-term gestation (Pasca et al., 2015; Qian et al., 2019; Kathuria et al., 2020).

In this study, we used a pair of isogenic (euploid and trisomic) iPSCs derived from an adult female with DS to generate iPSC-derived CS, following a recently published protocol (Madhavan et al., 2018). In addition to morphological and histological examination, we also performed single cell RNA sequencing (scRNA-seq) to characterize molecular alterations at the single cell level of resolution. While our CS contained seven major cell types, including radial glial cells (RGCs), intermediate precursors (IPCs), astrocytes, and inhibitory neurons, our transcriptomic analysis identified excitatory neuron (ExN) clusters as the most affected by trisomy. Specifically, our studies identified a cluster of cells corresponding transcriptionally to layer IV cortical neurons (ExN4) as the major dysregulated cell type affected by trisomy 21. ExN4 displayed profound developmental divergence from the corresponding euploid cluster, including many differentially expressed (DEX) genes and affected processes related to neuronal motility and establishment of cortical architecture. The dataset also revealed gene candidates in specific cell types that drive alterations in developmental trajectories.

We then performed a direct comparison of our scRNA-seq study to previous datasets generated from the same isogenic lines as well as from human postmortem brain tissue (Olmos-Serrano et al., 2016; Palmer et al., 2021; Nava et al., 2022). This comparison revealed that despite differences in technical approaches and the source of trisomic samples, there is a portion of shared HSA21 and non-HSA21 genes affected in all the studies. This analysis also identified transcriptomic divergence and distinct transcriptional profiles relating to the specific genetic background of the individual (sex, allelic composition). By comparing the current CS dataset to previously published studies, we demonstrate the benefit of using isogenic cell lines in uncovering consistent biological factors across studies and platforms.

## Materials and methods

### Generation of cortical spheroids

We received a pair of isogenic lines, consisting of a trisomic line (WC-24-02-DS-M) and a euploid control (WC-24-02-DS-B), as a generous gift from Anita Bhattacharyya’s lab at the University of Wisconsin, Madison. These lines were validated previously and deposited at WiCell^®^ Research Institute (Madison, WI, United States). IPSCs were passaged and cultured on Matrigel^®^ (Corning, New York, NY, United States) using mTeSR™ plus media (StemCell Technologies^®^, Vancouver, AB, Canada). Cells below passage 30 were used to generate CS. About 1.5 × 10^6^ trisomic and euploid iPSCs dissociated with accutase (StemCell Technologies, Vancouver, AB, Canada) were used to generate around 100 spheroids that were differentiated further into CS following a published protocol with modifications (Madhavan et al., 2018). Briefly, the dissociated cells were transferred to individual low-adherence V-bottom 96-well plates (S-Bio Prime, Hudson, NH, United States) in 150 μl TeSR5/6 media (StemCell Technologies^®^, Vancouver, AB, Canada) with 50 μM Rock inhibitor Y-27632 (Tocris BioScience, Bristol, United Kingdom), 5 μM Dorsopmorphin (Tocris BioScience, Bristol, United Kingdom) and 10 μM SB-431542 (Tocris BioScience, Bristol, United Kingdom). The same media without Rock inhibitor was used and changed daily for the next 5 days. On day six, the media was changed to spheroid media containing Neurobasal-A media supplemented with B-27 without vitamin A (Invitrogen/Life Technologies, Carlsbad, CA, United States), Glutamax (Invitrogen/Life Technologies, Carlsbad, CA, United States), and Penicillin/Streptomycin. Basic fibroblast growth factor (FGF-2, 20 ng/ml, R&D systems, Minneapolis, MN, United States) and epidermal growth factor EGF (10 ng/ml, R&D systems, Minneapolis, MN, United States) were added to the media on days 7–24. On day 25, spheroids were transferred to ultra-low attachment 24-well plates (Corning, New York, NY, United States) and 1% Geltrex (Invitrogen/Life Technologies, Carlsbad, CA, United States) was added to the media. Brain Derived Neurotrophic Factor (BDNF, 20 ng/ml, R&D systems, Minneapolis, MN, United States) and Neurotrophin-3 (NT-3, 20 ng/ml, R&D systems, Minneapolis, MN, United States) were used for neural differentiation between days 27 and 41. To expand the existing small population of oligodendrocytes in the spheroids, beginning on day 50, 10 ng/ml platelet-derived growth factor-AA (PDGF-AA, R&D systems, Minneapolis, MN, United States) and insulin-like growth factor-1 (IGF-1, R&D systems, Minneapolis, MN, United States) were supplemented to the media changes for 10 days. Between days 50 and 60, the media was supplemented with 40 ng/ml 3,3′,5-triiodothronine (T3, R&D systems, Minneapolis, MN, United States). The CS were maintained in spheroid media from day 70 until completion of the experiment with half-media changes every other day. Multiple, temporally overlapping spheroid cultures were generated to provide a constant source for sampling and analysis of developmental markers. The mycoplasma contamination test was performed regularly using PCR Mycoplasma Test Kit I/C (PromoCell^®^, Heidelberg, Germany).

### Single cell dissociation and capture

Cortical spheroids dissociation was performed on day 130 as described. Four CS generated in different wells were pooled per sample and dissociated with Worthington Papain dissociation system (Worthington Biochemical Corp., Lakewood, NJ, United States, Cat#: LK003150) following the protocol by the manufacturer. Prior to dissociation, we oxygenated the papain solution with 95% O_2_ and 5% CO_2_ to insure cell viability. The CS were first cut into small pieces and then dissociated in 20 units/ml papain and 0.005% DNase solution at 37°C with thorough constant agitation for 40 mins. The mixture was titrated with 5 ml pipette and the cell suspension was centrifuged at 300 g for 2 mins at room temperature. The pellet was resuspended with PBS containing 1% BSA. Cell viability and number was assessed using Tripan-Blue on Countess automatic cell counter (Invitrogen/Life Technologies, Carlsbad, CA, United States). Cell samples at a concentration of 1,000 cells/μl were submitted for a single cell capture. 10X Genomics Chromium^®^ single cell preparation system was used for cell capture following manufacturer’s protocol.

### Library preparation and sequencing

The synthesis of cDNA, cDNA amplification, and the preparation of the libraries were performed using the 10× Genomics Chromium Single Cell 3′ Library and Bead Kit (v3). according to manufacturer’s instruction. Sequencing was done on NovaSeq 6000 at the Single Cell Sequencing Core at Boston University School of Medicine.

### Read alignment

Fastq files containing pair-end reads of each sample were aligned to GRCh38 Genome Reference Consortium Human Reference 38 (hg38) and GENCODE annotation (v35) using Cellranger (v3.1.0) *count* function with default settings. Cellranger *aggr* function was then used to combine aligned and filtered count matrix from all samples.

### Bioinformatics analyses

#### Quality control

Cells with (1) number of detected genes greater than 1,000 and (2) percentage of reads mapped to mitochondrial genome between 1 and 10% were kept. After filtering cells, only protein coding genes in each cell were used for downstream analyses. Mitochondrial genes were removed. Data were normalized using *NormalizeData* function from the *Seurat* R software package with normalization method set to “LogNormalize” and scale factor as 10,000 (Stuart et al., 2019).

#### Dimension reduction and clustering

To perform dimension reduction and clustering, we first identified the top 2,000 highly variable genes (HVGs) using *FindVariableGenes* function from the *Seurat* R software package. The HVGs were scaled before being applied to principal component analysis (PCA) as input. Top 10 principal components (PCs) with the highest standard deviation were used to perform UMAP dimension reduction resulting in a 2D representation of the dataset. Clustering was done first by calculating the neighborhood of each cell with *FindNeighbors* function on the two UMAP coordinates with k parameter set to 15. Then, *FindClusters* function was called with resolution set to 0.15.

#### Differential expression analyses

We conducted differential expression (DEX) analyses using Seurat function *FindAllMarkers*. We took cells from one cell type and compared it to the rest of all the cells, using a binomial model. For any given comparison, we only considered genes that were expressed by at least 25% of cells in either population. Genes that exhibit adjusted *p*-values under 0.1 were considered statistically significant. The Database for Annotation, Visualization, and Integrated Discovery (DAVID) v6.8 was used for gene ontology (GO) analysis (Huang et al., 2009). Briefly, all statistically significant genes for each cell cluster were entered into the database and statistically significant biological processes associated with the gene lists were identified (FDR < 0.05). Biological processes were reported in order of fold enrichment, or the ratio of the DEX genes in the list involved in a particular process to the total number of genes that could be involved in that process in *Homo sapiens*.

#### Diffusion map

To generate diffusion map (DM) for all cells in the dataset, we first selected the top 500 HVGs and performed PCA as described in the previous section. The top 20 PCs were used to find the optimal sigma (σ) using function *find_sigmas* from R package Destiny with default parameters (Angerer et al., 2016). Then, the top 20 PCs were used as input in function *DiffusionMap*, with 2σ as the diffusion scale parameter and number of nearest neighbors (*k*) set as 100. To calculate DM for each individual cell type, the abovementioned procedure was followed with data within each cell type as input and *k* set to 25.

#### Pseudotime analysis

We used R package URD following recommended steps with minor adjustments based on the structure of the dataset (Farrell et al., 2018). Briefly, a subset of aRGC1 cells near the center of the DM were set as the root. The DM was flooded 100 times to establish the pseudotime axis. Tips of the DM were identified from the final stage of pseudo development. Biased random walks were then performed from each tip for 10,000 times. Lastly, a tree graph was built using *buildTree* function with default settings except threshold of *p*-value set to 0.05.

#### Principal graph analysis

To identify genes associated with different regions of DM, we first manually converted our dataset from a URD object into a monocle object (Cao et al., 2019). Then, the function *graph_test* from R package monocle3 was performed on the monocle object. Moran’s I greater than 0.3 and adjusted *p*-value less than 0.01 were used as threshold to identify genes associated with either trisomic or euploid cells on the DM.

#### Inter-genotype distance

To assess the genotypic differences in each cell type, we first calculated the Euclidean distances between each trisomic cell to each euploid cell within each cell type using *dist* function on DM, which were then averaged to get observed inter-genotype distance (oIGD). We then performed 1,000 permutations within each cell type. During each permutation, the genotype labels were randomized within each cell type, and an inter-genotype distance (eIGD) was calculated by the same process as oIGD. Lastly, a *p*-value was calculated for oIGD based on the distribution of eIGDs of the same cell type. To compare between cell types, oIGD and eIGDs of each cell type were normalized by dividing the average Euclidean distance between each unique pairs of euploid cells within the respective cell type.

### Immunohistochemistry

For immunohistochemistry (IHC), the CS were fixed overnight with 4% ice-cold paraformaldehyde, washed three times, 10 mins each, with PBS, and cryoprotected in 30% sucrose overnight. The spheroids were embedded in 30% sucrose/Optimal Cutting Temperature compound (OCT; Sakura Finetek, Torrance, CA, United States) at 1:1 ratio and sectioned at 12 μm. Sections were washed three times with PBS, blocked for 30 mins in PBS containing 0.1% Triton X-100 (PBST) and then incubated in a blocking solution containing 5% donkey serum in PBST for an hour at room temperature. Next, the sections were incubated with the primary antibodies diluted in the blocking buffer at 4°C overnight. The next day, the slides were washed three times with PBST for 10 mins each, followed by incubation with secondary antibody for an hour at room temperature. Then, the slides were washed three times with PBS for 10 mins and coversliped with ProLong™ Gold Antifade Mountant with DAPI (Thermo Fisher Scientific, Waltham, MA, United States).

The following primary antibodies were used: rabbit anti-CC3 (1:750, Cell Signaling, Cell Signaling, Danvers, MA, United States, cat. number: 9661-s); mouse anti-SATB2 (1:250, Abcam, cat. number: ab51520); rat anti-CTIP2 (1:400, Abcam cat. number: ab18465); rabbit anti-FOXG1 (1:250, Abcam cat. number: ab196868); goat anti-SOX2 (1:250 R&D Systems, Minneapolis, MN, United States, cat. number: AF2018), rabbit anti-TBR1 (1:250, Abcam, cat. number: Ab31940). All secondary antibodies were AlexaFluor conjugated, used at a dilution of 1:500 and obtained from LifeTechnologies.

### Confocal microscopy, imaging, and quantification

For each organoid, three to four regions of cut sections were imaged per spheroid using a Zeiss LSM 710 confocal microscope system (Carl Zeiss, Jena, Germany, GER) and z-stacks (1,024 × 1,024 pixels) were collected using a 20× or 40× objective lens. For markers of developing neurons (SATB2, CTIP2, and TBR1), the cortical plate regions imaged were located in the vicinity of the ventricular-like zones present in the spheroid sections that were identified by morphology and presence of positive cells. To analyze cortical layer markers that colocalized with individual nuclei (SATB2, CTIP2, TBR1, etc.), labeled cells in each z-stack were counted using the ACEq application, a “3-dimensional version” of the app that was designed to quantitatively assess markers across the z-stack and correct for overlap as described previously (Klein et al., 2022). This version can be publicly accessed through Zeldich lab website^1^ and has been validated previously against manual quantification of cell numbers (Klein et al., 2022). For the quantification of CC-3, regions were randomly chosen along the spheroid edge, away from the center of the spheroids. Since the individual cell quantification (as we did for the cortical markers) was not possible due to the presence of cell debris/apoptotic bodies, the optical density of antibody labeling was assessed and quantified using particle analyses function through the imageJ/FIJI (RRID:SCR_003070; Medalla et al., 2017). For the analyses, the threshold for the signal was set in the first field and subsequently applied to the rest of the fields of the same image. The percent of antibody-recognized area was calculated out of the total area covered by DAPI for each field. The counts were first averaged for each region of the sliced organoid and then the values were averaged for a value for each organoid and that were finally averaged to reflect the total number of the organoids per condition to calculate a representation of mean ± standard error of the mean.

### Statistical analyses and data presentation

For IHC experiments quantification, Graphpad Prism software was used for the plotting of the data and assessing statistical significance between the conditions. We used an unpaired two-tailed student’s *T*-test to compare the quantification of cortical layer markers in isogenic euploid and trisomic CS following IHC. For the measurements of the size of the organoids across different time points, one-way ANOVA with *post hoc* Tukey’s test was used.

Exact hypergeometric probability test was used to calculate the statistical significance of overlap between DEX genes of different datasets. Kolmogorov-Smirnov test was used to assess the distribution of cell density along pseudotime. All graphs related to bioinformatics analyses were generated with *ggplot2* R package except when noted otherwise (Wickham, 2009).

## Results

### Generation of euploid and trisomic cortical spheroids containing diverse cell lineages

**FIGURE 1:**
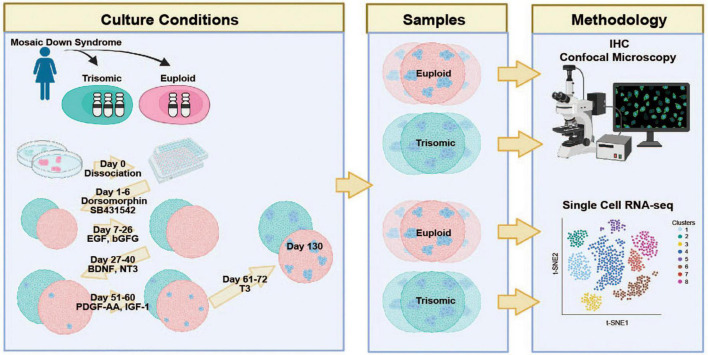
Schematic representation of the experimental protocol and study design Isogenic HSA21 euploid (red) and trisomic (blue) induced pluripotent stem cell (iPSC) lines derived from a woman with Down Syndrome were differentiated into cortical spheroids (CS) and analyzed at day 130 via IHC and scRNA-seq. Created with BioRender.com.

**FIGURE 2:**
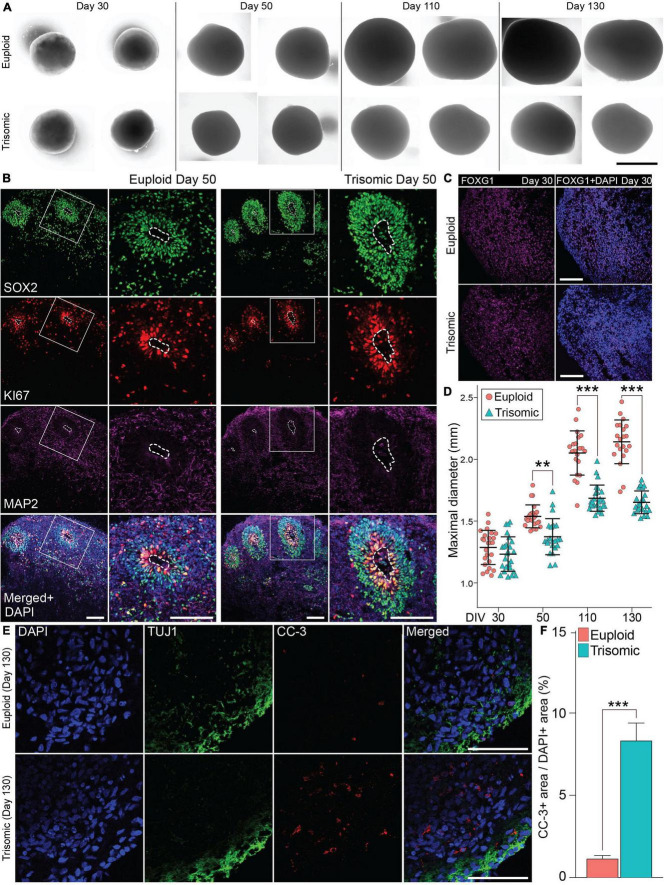
Generation and characterization of isogenic cortical spheroids (CS). **(A)** Bright field images of euploid and trisomic CS on day 30, 50, 110, and 130. Scale bar, 1 mm. **(B)** Immunohistochemistry (IHC) staining of SOX2 showing the presence of the rosette-like structures enriched with ki67 marker in euploid and trisomic CS on day 50. Scale bar, 100 μm **(C)** IHC staining with FOXG1 antibody on day 30 in euploid and trisomic CS. **(D)** Jitter plot showing the distribution of CS diameters on days 30 (euploid, *n* = 25; trisomic, *n* = 25), 50 (euploid, *n* = 23; trisomic, *n* = 22), 110 (euploid, *n* = 21; trisomic, *n* = 25), and 130 (euploid, *n* = 21; trisomic, *n* = 22). Euploid samples are represented by red circles, while trisomic samples are represented by blue triangles. The results are analyzed by one way ANOVA followed by Tukey’s multiple comparisons test. **(E)** IHC staining of CC-3 and TUJ1 in euploid and trisomic CS on day 130. Scale bar, 50 μm. **(F)** Bar graph showing the percentage of area with CC-3 IHC signal over the area with DAPI signal quantified through particles analysis via ImageJ s and analyzed using student *t*-test (euploid, *n* = 14; trisomic, *n* = 13). Error bar represents standard error. **p < 0.01; ***p < 0.001. The quantification results are generated from three independent differentiation experiments.

Following a recently published protocol (Madhavan et al., 2018), we generated dorsal forebrain fated CS from an isogenic pair of euploid (WC-24-02-DS-B) and trisomic (WC-24-02-DS-M) iPSCs derived from an adult female with DS that contained progenitors, neurons, astrocytes, and oligodendrocytes. We continuously cultured the CS until 130 days, when CS were transcriptomically profiled by scRNA-seq and subjected to immunohistochemistry (IHC) (Figures 1, 2A). We observed multiple rosette structures reminiscent of cortical ventricular zones (VZ) on day 50 enriched with the ectoderm and neural stem cell marker sry-box transcription factor 2 (SOX2) and nuclear protein ki67, a proliferation marker in both euploid and trisomic CS (Figure 2B). In addition, we also confirmed the forebrain identity of the isogenic CS by staining with forkhead box G1 (FOXG1) (Figure 2C). Following continuous culturing to allow neuronal and glial differentiation, we successfully verified the presence of neurons by microtubule associated protein 2 (MAP2) positivity and astrocytes by glial fibrillary acidic protein (GFAP) positivity starting from day 50, following by the emergence of oligodendrocytes confirmed by CC1 IHC. These cell types continued to mature and were present at day 130 (Supplementary Figures 1A,B).

### Trisomic cortical spheroids display a reduced cortical volume

The euploid and trisomic CS developed in a comparable manner as measured by spheroid diameter during early stages of differentiation (day 30), preceding the induction of cortical expansion by the application of the neurotrophins BDNF and NT3 (1,231 ± 19.8 μm, trisomic; 1,285 ± 19.9 μm, euploid; *p* = 0.45; Figures 2A,D). The size of the spheroids diverged upon the completion of the neurotrophin treatment at Day 50, as the diameter of trisomic CS was significantly smaller compared to euploid controls (1,372 ± 20.9 μm, trisomic; 1,536 ± 13.2 μm, euploid; *p* = 0.002, Figure 2D). The size differences became even more pronounced on day 110, when trisomic organoids measured at an average of 1,684 ± 15.1 μm, while euploid organoids measured 2,050 ± 25.6 μm (*p* < 0.001, Figures 2A,D). On day 130, trisomic organoids measured at an average of 1,650 ± 13.1 μm, while euploid organoids at 2,140 ± 25.3 μm (*p* < 0.001, Figure 2D). The difference in size is consistent with smaller size of embryonic bodies and brain organoids reported by other groups (Tang et al., 2021) and is in line with reduced cortical volume in individuals with DS (Wisniewski, 1990; Baburamani et al., 2020).

We hypothesized that apoptosis may be an underlying cause of the consistent decrease in size of the trisomic CS. Therefore, we examined markers of apoptosis using IHC of cleaved caspase-3 (CC-3). No statistical differences in area of CC-3 expression (normalized to the area occupied by DAPI) were detected on day 30 between euploid CS (5.31 ± 1.45%) and trisomic CS (6.93 ± 2.2%) (*p* < 0.57; Supplementary Figure 2A). This in line with comparable size measurements of CS at this time point. However, we found increased CC-3 IHC signal in trisomic CS at day 90 (10.3 ± 2.1%) compared to euploid CS (4.7 ± 0.9%) (*p* < 0.038; Supplementary Figure 2B). On day 130, CC-3 labeling was found in 8.4 ± 1.1% of trisomic CS area whereas only 1.2 ± 0.2% of CC-3+ area was found in euploid CS (*p* < 0.001; Figures 2E,F). These results suggest that the reduced size of the trisomic CS can be attributed at least in part to increased cell death.

### scRNA-seq analysis unravels alteration in neural development in trisomy 21 cortical spheroids

To further characterize the CS, we performed scRNA-seq analysis on Day 130 (Figure 1). Two samples were collected for each genotype and each sample consisted of a pool of four CS from the same differentiation experiment (eight spheroids per genotype). The samples were processed following 10X Genomics scRNA-seq protocol and an estimated number of 8,890 cells were captured. To confirm the reproducibility of sample preparation and sequencing, we compiled reads by sample and compared the genomic coverage across samples in five million base pair windows across the entire genome. We observed identical patterns of genomic coverage across the four samples, except on HSA21 where reads from the trisomic CS displayed an elevated level of disturbance compared to euploid samples (Supplementary Figure 3). To further confirm the effect of trisomy at the level of individual samples, we performed differential gene expression (DEX) analysis between trisomic and euploid samples using *DESeq2* program (Love et al., 2014). As expected, in trisomic samples we observed a much greater number of upregulated than downregulated genes on HSA21. In contrast, the numbers of up- or downregulated genes on the other autosomes were comparable (Supplementary Figure 3). After quality control (QC), 6,093 cells were kept for downstream analysis, of which 3,077 were euploid and 3,016 were trisomic. At the level of individual cells, we detected an average of over 3,000 genes in each cell with an average read depth (UMI) around 10,000 (Supplementary Figure 4A). All cells that passed QC had no more than 10% and no less than 1% of total reads mapped to mitochondrial genome (Supplementary Figure 4A). We then performed dimension reduction and depicted the transcriptome from each cell in 2D space using UMAP (Becht et al., 2018). No batch effect or overt differences between trisomic and euploid samples were observed (Supplementary Figures 4B–D).

**FIGURE 3:**
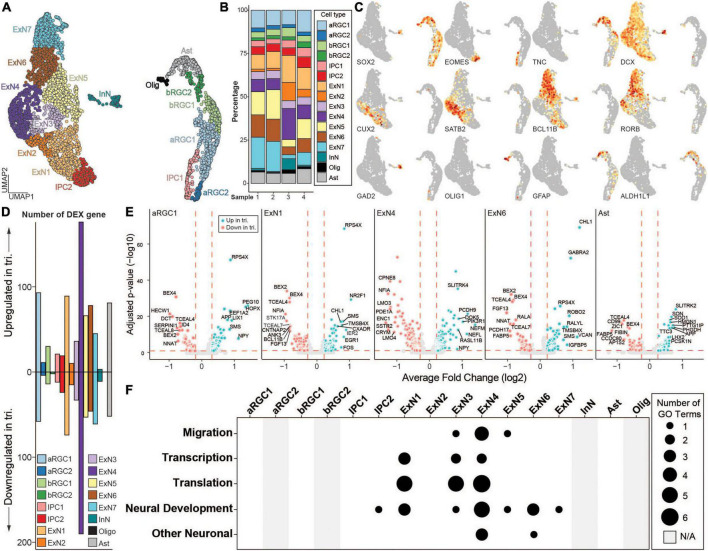
scRNA-seq analysis of isogenic euploid and trisomic cortical spheroids (CS) at day 130. **(A)** UMAP representation of scRNA-seq data collected from two euploid and two trisomic CS samples. Colors represent identified cell types. ARGC, apical radial glial cell; bRGC, basal radial glial cell; IPC, intermedial progenitor cell; ExN, excitatory neuron; InN, inhibitory neuron; Ast, astrocyte; Olig, oligodendrocyte. **(B)** Bar graph showing the percentage of each identified cell type in each sample. Colors represent same cell types as in panel **(A)**. Sample 1 and 2 are euploid, whereas 3 and 4 are trisomic. **(C)** UMAP as in panel **(A)** showing gene expression levels of canonical markers. Colors represent normalized gene expression level (norm. exp.). **(D)** Bar graph showing the number of up- or down-regulated differentially expressed (DEX) genes in trisomic vs. euploid single cells by cell type. Colors represent same cell types as in panel **(A)**. **(E)** Volcano plots showing DEX genes in cell types. Five cell types with the highest numbers of DEX genes are shown. Colors represent genotype (euploid, red; trisomic, blue). Vertical red dashed lines represent average log2 fold change of –0.25 or 0.25. Horizontal red dashed lines represent adjusted *p*-value of 0.1. Eu, euploid; tri, trisomy. **(F)** Dot plot showing the number of enriched gene ontology (GO) terms in each cell type. Enriched GO terms are grouped into five categories of “Migration”, “Transcription”, “Translation”, “Neural Development” and “Other Neuronal.” Size of the dot represents number of enriched GO terms. Gray bar represents cell types where no DEX genes were identified and thus not applicable (N/A) to the GO analysis.

Next, we performed unsupervised clustering following *Seurat* v3 pipeline (Stuart et al., 2019) and identified 16 clusters representing seven major cell types (Figure 3A). The major cell types include apical radial glia cells (aRGC), basal radial glial cells (bRGC), intermediate progenitor cells (IPC), astrocytes (Ast), oligodendrocytes (Olig), inhibitory neurons (InN), and excitatory neurons (ExN). All cell types were present in each of the four samples (Figure 3B). No statistically significant difference was found comparing the percentages of each cell type between the two genotypes using two sample *t*-test (all *p*-values > 0.05). Each cell type expressed canonical marker genes including *SOX2* (RGCs), eomesodermin (*EOMES*) (IPCs), cut like homeobox (*CUX2*, layer II/III ExN), special AT-rich sequence-binding protein (*SATB2*, layer II-IV ExN), RAR related orphan receptor B (*RORB*, layer IV ExN), BAF chromatin remodeling complex subunit (*BCL11B*, layer V ExN), glutamate decarboxylase 2 (*GAD2*, InN), *OLIG1* (Olig), as well as astrocytic markers, *GFAP* and aldehyde dehydrogenase 1 family, member L1 (*ALDH1L*) (Figure 3C). We performed DEX analysis between the two genotypes in each cell type that we identified. The majority of the DEX genes were found in the ExN clusters, with the number of DEX genes highest in ExN4 (Figure 3D). Of the five cell types with the most DEX genes, four were excitatory neuron clusters (Figures 3D,E and Supplementary Figure 4E).

We then performed gene ontology (GO) analysis to identify biological processes that are significantly enriched in each one of the cell types, using up and down regulated genes and a threshold of FDR < 0.05 to identify significantly affected biological processes (Figure 3F and Supplementary Figure 5). These biological processes were further categorized into five groups: “migration”, “transcription”, “translation”, “neural development” and “other neuronal”. Consistent with the DEX analysis, excitatory neuron cell types showed the most significant enrichment with the highest number of enriched terms in all five categories, suggesting that DEX genes in ExN cell types converged on similar biological processes. In contrast, DEX genes from neural progenitor cell types (i.e., aRGC1, bRGC1, and IPC1) as well as Ast did not show any enrichment and thus had no functional convergence, even though the number of DEX genes was comparable to those from ExNs (Figures 3D,F).

**FIGURE 4:**
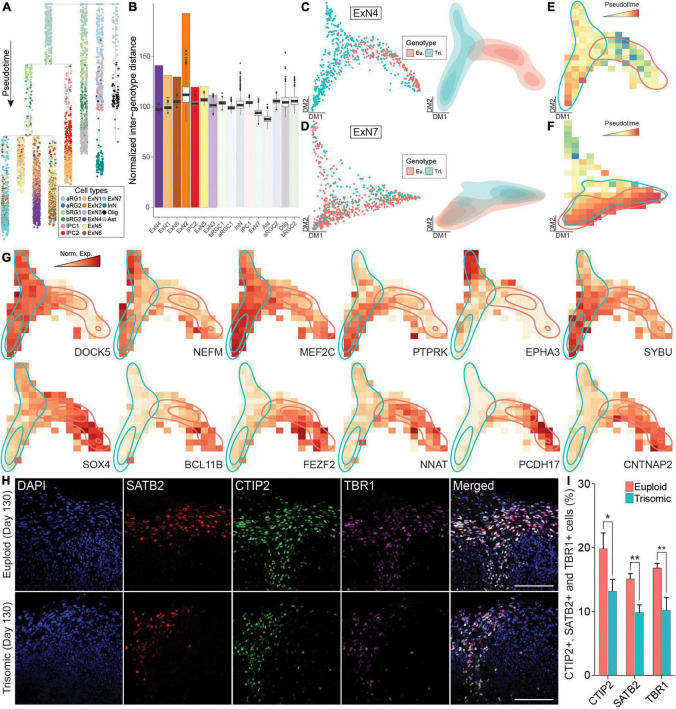
Pseudotime analysis of scRNA-seq data from euploid and trisomic cortical spheroids (CS) at day 130**. (A)** Dendrogram showing single cells along pseudotime. Branches on dendrogram signify divergence in transcriptome profiles. Colors represent cell types. **(B)** Bar graph showing observed normalized inter-genotype distance (IGD) in each cell type. Box plots on top of each observed IGD show estimated IGDs from 1,000 permutations. Cell types are arranged by statistical significance of observed IGC. The first six cell types on the graph are statistically significant (*p*-value < 0.001). **(C)** Diffusion map (left panel) showing single cells and density plot (right) showing the distribution of single cells from ExN4 cell type. Colors represent genotype (eu., euploid, red; tri., trisomic, blue). **(D)** Diffusion map (left panel) showing single cells and density plot (right) reflecting the distribution of single cells from ExN7 cell type. Colors represent genotype (euploid, red; trisomic, blue). **(E)** Raster plot showing pseudotime in diffusion map space of ExN4 as in panel **(C)**. Colors represent pseudotime. Regions with high density of euploid (red) or trisomic (blue) cells are outlined. **(F)** Raster plot showing pseudotime in diffusion map space of ExN7 as in panel **(D)**. Heatmap colors represent pseudotime. Regions with high density of euploid (red) or trisomic (blue) cells are outlined. **(G)** Raster plot showing expression levels in diffusion map space as in panel **(C)** of genes specifically associated with trisomic or euploid cells in ExN4. Heatmap colors represent normalized gene expression levels (norm. exp.). Regions with high density of euploid (red) or trisomic (blue) cells are outlined. **(H)** IHC staining with anti- SATB2, anti-CTIP2, and anti-TBR1 antibodies in euploid and trisomic CS on day 130. **(I)** Bar graph showing the percentage of cells expressing CTIP2, SATB2 or TBR1 IHC signal calculated by quantifying the ratio of number of CTIP2+, SATB2+, or TBR1+ over total number of cells stained with DAPI and multiplied by 100%. The quantification is performed using ACEq application and analyzed using student *t*-test (euploid, *n* = 16; trisomic, *n* = 15). Error bar represents standard error. *p < 0.05; **p < 0.01. The quantification results are generated from three independent differentiation experiments; Scale bar, 100 μm.

We next performed pseudotime analysis to establish the differentiation trajectory for all cells (Figure 4A and Supplementary Figure 6). To quantify the difference driven by trisomy for each cell type, we calculated the average Euclidean distance on diffusion maps (DMs) between each trisomic cell and each euploid cell within the same cell type and used it as a presentation of transcriptome divergence between the genotypes (Supplementary Figure 7). We term this value “observed inter-genotype distance” or oIGD. To identify statistically significant oIGD, we randomized the genotype assignment 1,000 times within each cell type and calculated a distribution of estimated IGD (eIGD). By comparing oIGDs to eIGDs, we identified seven cell types with statistically significant oIGDs indicating a significant transcriptomic divergence between the genotypes. These included – in the order of most significant to least significant - ExN4, ExN1, ExN6, ExN2, IPC2, ExN5, and ExN3 (Figure 4B). In contrast, other cell types (including ExN7) did not have significant oIGDs and thus did not show genotypic divergence in our dataset. We then further compared ExN4, the cell type with the greatest genotypic divergence (Figure 4C), to ExN7 that displays no genotypic divergence (Figure 4D). In line with the IGD analysis, the cell density and pseudotime distribution of trisomic and euploid cells were significantly different on the DM and non-overlapping (Figures 4C,E). In contrast, trisomic and euploid cells from ExN7 completely overlapped in cell density distribution (Figures 4D,F). Together, this indicates that the development of some ExN clusters, including ExN4, is more severely impacted by trisomy 21 than other cell types.

To identify genes driving the differences between the trisomic and euploid ExN4 cells, we performed principal graph analysis (PGA) (Cao et al., 2019). Superior to standard DEX analyses that are based solely on expression levels, PGA identifies genes not only by up- or downregulation between conditions, but also with non-random patterns along pseudotime, which we refer to as “association.” We identified ten genes that were specifically associated with trisomic cells in ExN4 including ephrin type-A receptor 3 (*EPHA3)* and myocyte enhancer factor 2C (*MEF2C*), which have been shown to function in motility and migration during neural development (Figure 4G, Table 1, and Supplementary Figure 8). Among the genes unassociated with trisomic cells in ExN4 were several neuronal transcription factors such as *BCL11B* and *FEZF2*, as well as protocadherins, *PCDH17* and *PCDH19*, all of which play key roles in cortical development (Figure 4G, Table 1, and Supplementary Figure 8; Chang et al., 2018; Du et al., 2021; Hoshina et al., 2021; Sadegh et al., 2021; Tsyporin et al., 2021). To validate the findings from scRNA-seq, we examined the protein expression of deep and superficial cortical layer markers, BCL11B (CTIP2) and SATB2, respectively in CS. Our analysis showed a significant decrease in the percentage of trisomic cells expressing CTIP2 (trisomic, 13.4 ± 1.9%; euploid, 20 ± 2.4%; *p* < 0.04) as well as SATB2 (trisomic, 10 ± 1.1%; euploid, 15.34 ± 0.8%; *p* < 0.0015) at day 130 of differentiation. We also performed IHC staining for TBR1, a newborn neuron marker, and found 10.4 ± 2% of trisomic cells were positive for the protein whereas 17 ± 0.8% of euploid were positive (*p* < 0.0008) at day 130 of differentiation (Figures 4H,I). The same reduction in the percentage of trisomic cells expressing these markers compared to euploid cells was observed on day 90 of differentiation: CTIP2 (trisomic, 21 ± 4.4%; euploid, 39.4 ± 3%; *p* < 0.011), SATB2 (trisomic, 3.6 ± 1.6%; euploid, 20.4 ± 3.1%; *p* < 0.0012), and TBR1 (trisomic, 17 ± 5.2% euploid, 33.4 ± 1.6%; *p* < 0.032; Supplementary Figures 9A,B). These data suggest that abnormal neurogenesis of excitatory neurons may also contribute to the reduction in trisomic CS volume, which is reminiscent of the reduction of cortical volume in individuals with trisomy 21.

**TABLE 1:** Genes from the principal graph analysis of ExN4 that are associated or unassociated with the trisomic genotype.

Gene	Full name	Description	References
EPHA3	EPH receptor A3	Receptor tyrosine kinase implicated in cell-cell adhesion, cell migration, and axon guidance	Yun et al., 2003; Brennaman et al., 2014
DCLK3	Doublecortin like kinase 3	Predicted protein of the doublecortin superfamily	Mullins et al., 2021
TGFB2	Transforming growth factor beta 2	Secreted ligand of TGFβ proteins; Involved in SMAD signaling	Wang et al., 2020
NEFM	Neurofilament medium chain	Intermediate filament; plans a role in intracellular transport in axons and dendrites	Bergström et al., 2021
PIK3R1	Phosphoinositide-3-kinase regulatory subunit 1	Plays an important role in the metabolic actions of insulin	Jia et al., 2020
DOK5	Docking protein 5	Participates in RET-mediated neurite outgrowth	Liu et al., 2010
PTPRK	Protein tyrosine phosphatase receptor type K	Regulates cell growth differentiation and mitosis	Shen et al., 1999
MEF2C	Myocyte enhancer factor 2C	Transcription factor important for neocortical development	Li et al., 2018
RPS4X	Ribosomal protein S4 X-linked	Ribosome invlolved in local translation in axon	Shigeoka et al., 2019
SYBU	Syntabulin	Component of kinesin motor complex for anterograde axonal transport	Bereczki et al., 2018
SOX4	SRY-box transcription factor 4	Transcription factor important for neurodevelopment	Shim et al., 2012; Da Silva et al., 2021
PBX1	PBX homeobox 1	Transcription factor implicated in regional patterning of the brain	Ypsilanti et al., 2021
MEIS2	Meis homeobox 2	Transcription factor essential for development	Matsuda et al., 2019
VCAN	Versican	Proteoglycans of extracellular matrix	Armstrong et al., 2020
IGSF3	Immunoglobulin superfamily member 3	Immunoglobulin-like membrane protein involved in neuronal morphogenesis	Usardi et al., 2017
ENC1	Ectodermal-neural cortex 1	A member of the kelch family; interacts with actin	García-Calero and Puelles, 2005
FEZF2	FEZ family zinc finger 2	Transcription factor essential for projection neuron development	Sadegh et al., 2021; Tsyporin et al., 2021
PCDH19	Protocadherin 19	A member of protocadherin subclass of the cadherin superfamily	Hoshina et al., 2021
DUSP4	Dual specificity phosphatase 4	Mitogen Activated Protein Kinase (MAPK) inhibitor	Kirchner et al., 2020
CNTNAP2	Contactin associated protein 2	Cell adhesion molecule of the neurexin family	Klibaite et al., 2022
LMO7	LIM domain 7	Protein-protein interaction	Lencer et al., 2017
SSTR2	Somatostatin receptor 2	Regulates neuronal calcium signaling	Agoglia et al., 2021
PDE1A	Phosphodiesterase 1A	Important cellular signal transduction molecule	Stoner et al., 2014
NFIA	Nuclear factor I A	Transcription factor that regulates central nervous system development	Ogura et al., 2022
BCL11B	BAF chromatin remodeling complex subunit BCL11B	Transcription factor regulating development of cortical projection neurons	Du et al., 2021
NNAT	Neuronatin	Proteolipid involved in the regulation of ion channels	Kanno et al., 2019
TOX	Thymocyte selection associated high mobility group box	Transcription factor controlling proliferation of neural stem cells	Artegiani et al., 2017
CRYM	Crystallin Mu	Binds to thyroid hormone and regulates neurodevelopment by binding to thyroid hormone	Hallen and Cooper, 2017
PCDH17	Protocadherin 17	A member of protocadherin subclass of the cadherin superfamily important for establishing cell-cell connections in the brain	Chang et al., 2018
CPNE8	Copine 8	Calcium-dependent membrane-binding protein	Florentinus-Mefailoski et al., 2021

### Comparison with other transcriptomic studies reveals common and diverged gene dysregulation in trisomy 21 cortical spheroids

**FIGURE 5:**
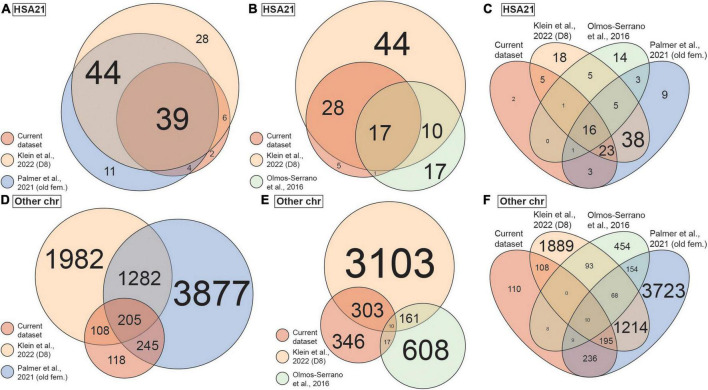
Overlap of differentially expressed (DEX) genes between the current and published datasets. **(A)** Venn diagram showing overlap of DEX genes on chromosome 21 (HSA21) between the current, Palmer et al. (2021) and Klein et al. (2022) datasets. Circle size represents number of DEX genes. **(B)** Venn diagram showing overlap of DEX genes on HSA21 between the current, Olmos-Serrano et al. (2016) and Klein et al. (2022) datasets. Circle size represents number of DEX genes. **(C)** Venn diagram showing overlap of DEX genes on HSA21 between all four datasets as in panels **(A,B)**. **(D)** Venn diagram showing overlap of DEX genes on all chromosomes except HSA21 between the current, Palmer et al. (2021) and Klein et al. (2022) datasets. Circle size represents number of DEX genes. **(E)** Venn diagram showing overlap of DEX genes on all chromosomes except HAS21 between the current, Olmos-Serrano et al. (2016) and Klein et al. (2022) datasets. Circle size represents number of DEX genes. **(F)** Venn diagram showing overlap of DEX genes on all chromosomes except HSA21 between all four datasets as in panels **(D,E)**. Colors represent datasets. Only data of old female (old fem.) samples are included from Palmer et al. (2021) dataset and only data of day 8 WC-24-02-DS (D8) iPSC cultures are included from Klein et al. (2022). Size of the text in all panels represents number of DEX genes.

To put our findings in a broader context, we compared our current scRNA-seq dataset to three previously published DS transcriptomic datasets, including bulk RNA-seq data of brain-like NPCs differentiated from iPSCs (Klein et al., 2022), bulk microarray data of postmortem human brain (Olmos-Serrano et al., 2016), and scRNA-seq data of postmortem brains (Palmer et al., 2021). It is worth noting that Klein et al. (2022) dataset was generated from the same isogenic line [WC-24-02-DS-B (euploid) and WC-24-02-DS-M (trisomic)] we used to generate the CS in our current study. To ensure a meaningful comparison, only data from iPSCs 8 days after induction (representing neural progenitor cells) from Klein et al. (2022) dataset were used. From Palmer et al. (2021) study, we analyzed both the full dataset which consisted of samples from multiple age groups and both sexes (“all”), as well as a selection of the older female samples (“old fem.”) to examine the potential confounds from mixing different biological sexes. We first investigated the DEX genes from each study located on chromosome 21 (HSA21) and found statistically significant overlaps between every pair of datasets (*p* < 0.001; Figures 5A,B). Particularly, 45 (88%) and 43 (84%) out of the 51 HSA21 DEX genes from our scRNA-seq dataset were also DEX in the Palmer et al. (2021) and Klein et al. (2022) (old fem.) datasets, respectively. In addition, 18 (35%) of 51 the HSA21 DEX genes from our dataset were also DEX in the Olmos-Serrano et al. (2016) dataset. Remarkably, we found 16 DEX genes on HSA21 that were present in all four datasets (Figure 5C and Table 2).

**FIGURE 6:**
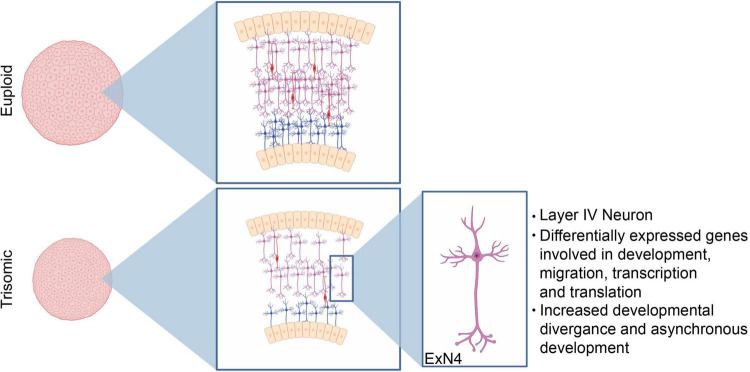
Neurodevelopmental changes driving decreased trisomic spheroid size. Our data demonstrates that the consistent decrease in volume of the trisomic cortical spheroids primarily stems from perturbed neurodevelopmental processes including decreases in excitatory neuron production. Specifically, our bioinformatic analysis identifies a cluster of excitatory neurons with a transcriptional signature identifying them as layer IV cortical neurons as the most affected cell type.

**TABLE 2:** Sixteen differentially expressed (DEX) genes on HSA21 that were present in all four datasets.

Gene	Full name	Description	References
SOD1	Superoxide dismutase	A cellular antioxidant, breaks down reactive oxygen species	Rosen et al., 1993; Milani et al., 2011
PCNT	Pericentrin	Plays a role in the organization of the centromeres and mitotic spindle formation	Chen et al., 1996
PFKL	Phosphofructokinase	Participates in glucose metabolism	Levanon et al., 1989
GART	Trifunctional purine biosynthetic protein adenosine-3	Important in the biogenesis of purines	Gnirke et al., 1991
PRMT2	Protein arginine methyltransferase 2	Plays an important role in the metabolism and formation of nuclear pre-mRNA	Scott et al., 1998
PDXK	Pyridoxal kinase	Plays a role in vitamin B6 metabolism	Hanna et al., 1997
SON	SON DNA and RNA Binding Protein	Splicing co-factor contributing to efficient splicing of cell cycle regulators	Ahn et al., 2011
ITSN1	Intersectin 1	Regulates endocytic trafficking and actin polymerization	Guipponi et al., 1998
CRYZL1	Crystallin zeta like 1	Regulates glucose metabolism and lipogenesis.	Kim et al., 1999
BRWD1	Bromodomain and WD repeat domain containing 1	Participates in a formation of multicomplex proteins and participates in epigenetic regulation	Huang et al., 2003; Mandal et al., 2018
TMEM50B	Transmembrane protein 50B	Predicted to regulate late endosome and multivesicular body formation and sorting	Moldrich et al., 2008; Kong et al., 2014
MRPS6	Mitochondrial ribosomal protein S6	Participates in the protein synthesis within the mitochondrion	Oviya et al., 2021
PttG1IP	Pituitary tumor-transforming gene 1 protein-interacting protein	Interacts with a proto-oncogene, PTTG1 and plays a role in cancer	Chien and Pei, 2000
IFNAR1	Interferon alpha and beta receptor subunit 1	A part of the interferon pathway. Upon activation stimulates Janus protein kinase	Novick et al., 1994; Shemesh et al., 2021
IFNAR2	Interferon alpha and beta receptor subunit 2	A part of the interferon pathway. Upon activation stimulates Janus protein kinase and controls STAT phosphorylation	Shemesh et al., 2021
USP16	Ubiquitin specific peptidase 16	Deubiquitinating enzyme participating in the metaphase to anaphase transition in mitosis	Puente et al., 2003

We next examined the DEX genes on chromosomes other than HSA21 (non-HSA21). Here, 46 and 67% of the DEX genes from our dataset overlapped with those identified in the Palmer et al. (2021) and Klein et al. (2022) datasets (old fem.), respectively, and both overlaps were statistically significant (*p* < 0.001; Figure 5D). About 4% of the DEX genes from our dataset overlapped with the Olmos-Serrano et al. (2016) dataset, which was not statistically significant (*p* = 0.449; Figure 5E). Despite the technical and biological differences of the datasets, we were able to identify 10 non-HSA21 genes that were DEX in all four datasets (Figure 5F), some of which have previously been linked to DS or other neurodevelopmental phenotypes in DS (Table 3). To assess the influence of variability in individual genome on DEX gene discovery, we repeated the analyses replacing Olmos-Serrano et al. (2016) dataset with Palmer et al. (2021) (all) dataset (Supplementary Figures 10A–D). We first observed that Palmer et al. (2021) contained fewer unique HSA21 DEX genes (16 of 111, 14.4%) than Olmos-Serrano et al. (2016) (17 of 45, 37.8%), when compared to our dataset and Klein et al. (2022; Supplementary Figure 10A). As expected, most HSA21 DEX genes (95 of 98, 96.9%) from Palmer et al. (2021) (old fem.) were also DEX in Palmer et al. (2021) (all) (Supplementary Figure 10B). Interestingly, when we compared non-HSA21 DEX genes from Palmer et al. (2021) (all) to other datasets, we found substantially more unique DEX genes. In fact, 6031 (71.2%) non-HSA21 DEX genes were unique to Palmer et al. (2021) (all) when compared to our dataset and Klein et al. (2022) dataset (Supplementary Figure 10C). In addition, 5,429 (64.1%) non-HSA21 DEX genes were also unique in Palmer et al. (2021) (all) even with Palmer et al. (2021) (old fem.) included in the analysis (Supplementary Figure 10D). These observations suggest that multiple sample sources and variability in individual genomes contribute significantly to transcriptomic differences.

**TABLE 3:** Genes that are differentially expressed (DEX) in common across all four datasets: Olmos-Serrano et al. (2016); Palmer et al. (2021), and Klein et al. (2022), and the current scRNA-seq dataset generated for this study.

Gene	Full name	Description	References
GRIK3	Glutamate Ionotropic Receptor Kainate Type Subunit 3	Glutamate receptors are the predominant excitatory neurotransmitter receptors in the mammalian brain and are activated in a variety of normal neurophysiologic processes. This gene product belongs to the kainate family of glutamate receptors, which are composed of four subunits and function as ligand-activated ion channels	Shibata et al., 2006
NFE2L2	NFE2 Like BZIP Transcription Factor 2	Transcription factor which is a member of a small family of basic leucine zipper (bZIP) proteins. The encoded transcription factor regulates genes which contain antioxidant response elements (ARE) in their promoters; many of these genes encode proteins involved in response to injury and inflammation	Moi et al., 1994; Lanzillotta et al., 2021
SEMA5B	Semaphorin 5B	This gene encodes a member of the semaphorin protein family which regulates axon growth during development of the nervous system	Adams et al., 1996
POU6F2	POU Class 6 Homeobox 2	The POU family members are transcriptional regulators, many of which are known to control cell type-specific differentiation pathways	Fiorino et al., 2016
HECW1	HECT, C2 and WW domain containing E3 ubiquitin protein ligase 1	Predicted to enable ubiquitin protein ligase activity	Miyazaki et al., 2004
BDNF	Brain derived neurotrophic factor	During development, promotes the survival and differentiation of selected neuronal populations of the peripheral and central nervous systems. Participates in axonal growth, pathfinding and in the modulation of dendritic growth and morphology. Major regulator of synaptic transmission and plasticity at adult synapses in many regions of the CNS	Maisonpierre et al., 1991; Bimonte-Nelson et al., 2003
ARL4D	ADP ribosylation factor like GTPase 4D	ADP-ribosylation factor 4D is a member of the ADP-ribosylation factor family of GTP-binding proteins	Smith et al., 1995
JUND	JunD proto-oncogene, AP-1 transcription factor subunit	The protein encoded by this intronless gene is a member of the JUN family, and a functional component of the AP1 transcription factor complex. This protein has been proposed to protect cells from p53-dependent senescence and apoptosis	Labudova et al., 1998; Weitzman et al., 2000
COMT	Catechol-*O*-methyltransferase	Catechol-*O*-methyltransferase catalyzes the transfer of a methyl group from *S*-adenosylmethionine to catecholamines, including the neurotransmitters dopamine, epinephrine, and norepinephrine. This *O*-methylation results in one of the major degradative pathways of the catecholamine transmitters	Gustavson et al., 1982; Craddock et al., 2006
PRKX	Protein kinase X-linked	This gene encodes a serine threonine protein kinase that has similarity to the catalytic subunit of cyclic AMP dependent protein kinases. The encoded protein is developmentally regulated and may be involved in renal epithelial morphogenesis. This protein may also be involved in macrophage and granulocyte maturation	Li et al., 2005

## Discussion

In this study, we generated CS from isogenic iPSC lines derived from an adult female with DS to examine changes in early neurodevelopment at cellular resolution. The CS we generated as a model of dorsal forebrain development recapitulate the environment of fetal human neocortex at mid-gestation (Qian et al., 2019), including major cell types such as RGC, IPC, ExN, InN, and glial cells. This allows for a detailed examination of aberrant neurodevelopmental processes not only at a cellular level but also without the confound of multiple genetic backgrounds in a mixed sample set. Importantly, our trisomic CS captured one of the most salient features of neurodevelopment in DS that is thought to underlie the development of the intellectual disability in DS, a decrease in volume, which is reminiscent of the decreased cortical volume in brains of individuals with DS (Wisniewski, 1990; Golden and Hyman, 1994; Pinter et al., 2001; McCann et al., 2021). While we identified increased apoptosis as one of the contributing factors to the decreased volume, we hypothesized that changes in neurogenesis and neuronal differentiation, another well-defined phenotype in DS, also contributed to the smaller size of the CS (Schmidt-Sidor et al., 1990; Guidi et al., 2008; Larsen et al., 2008).

Both histological and scRNA-seq analyses point specifically to changes in excitatory neuron development in the trisomic CS. IHC analysis identifies a significant decrease in percentage of TBR1+ neurons at day 90 and 130 indicating that there are fewer newly differentiated neurons over an extended period during which neurons are born *in vitro*. There were also significant decreases in BCL11B (CTIP2) expression, a marker of mature neocortical layer V pyramidal neurons, and SATB2 a marker of layer II-IV neocortical excitatory neurons at the time points we tested. The decrease in neuronal marker gene and protein expressions indicate that cell populations in trisomic CS resembling both upper and deep layer neurons in the brain appear to be affected, supporting previous findings in DS-derived organoids (Tang et al., 2021). Using IGD, we identified transcriptional divergence between genotypes within multiple neuronal cell types and found that ExN cell types in general were most severely affected by HSA21 trisomy. In fact, six of the seven cell types with statistically significant IGD are ExN, consistent with the observation that four out of five cell types with the most DEX genes were ExN. Additionally, 47 of the 48 significantly enriched GO terms identified by our analyses were enriched in ExN cell types.

Although changes in neurogenesis have been described previously, the underlying molecular mechanism of these changes is unknown (Tang et al., 2021). In our study, we identified ExN4 as the most severely affected ExN cell type, as it had the largest number of DEX genes and the most significant inter-genotype distance among all cell types. Therefore, we focused our analysis on ExN4 to further understand molecular changes due to HSA21 in a cell type specific manner. Firstly, ExN4 expressed *SATB2* and *RORB4*, the combination of which indicate that these cells share transcriptomic signatures with layer IV excitatory neurons in human neocortex. The fact that ExN4 is the most profoundly affected cell type in our analysis may suggest that layer IV neocortical excitatory neurons in individuals with DS are also under higher pathological risk than other cell types during fetal development, supporting previous findings (Tang et al., 2021). Secondly, pseudotime analysis also indicated that there were changes in developmental trajectory of the trisomic cells. This developmental asynchrony between the two genotypes of ExN4 cells may be the result of transcriptional dysregulation leading to abnormal maturation. Indeed, based on our PGA results, several genes were associated strongly with the trisomic ExN4 cells while other genes were strongly unassociated with trisomic ExN4 cells. For instance, *BCL11B* and *Family Zinc Finger 2* (*FEZF2*), which are important transcription factors during fetal brain development, are unassociated with trisomic ExN4 cells. Both BCL11B and FEZF2, while commonly recognized as markers for layer V pyramidal neurons in postnatal neocortex, have transient broader expression patterns during fetal development in immature neurons (Du et al., 2021). During development, BCL11B is essential for the formation and maintenance of synapses (Simon et al., 2012) and its deficiency is associated with intellectual deficits, developmental delay and speech impairment (Punwani et al., 2016; Lessel et al., 2018). FEZ2F is expressed in postmitotic neurons and it regulates the acquisition of cell identity and specification in cortical projection neurons through the repression of alternative neuronal fate genes (Chen et al., 2005; Molyneaux et al., 2005; Shim et al., 2012; Tsyporin et al., 2021). Therefore, our observation that *BCL11B* and *FEZF2* are strongly unassociated with trisomic ExN4 cells may indicate a premature consolidation of transcriptional programs due to HSA21 trisomy. The premature shutdown of transcriptional program may in turn lead to alterations in the development of individual neurons, which may then manifest either in programmed cell death as we observed earlier, or in changes in neural plasticity and connectivity as suggested by previous human (Suetsugu and Mehraein, 1980; Ferrer and Gullotta, 1990; Medalla et al., 2017) and mouse (Dierssen et al., 2003; Belichenko et al., 2004; Pérez-Cremades et al., 2010) studies.

Lastly, among the genes strongly associated with the trisomic genotype in ExN4 are *EPHA3*, a gene critical for cytoskeleton organization, migration and cell adhesion of neural cells during nervous system development (Rudolph et al., 2010; Javier-Torrent et al., 2019) and *MEF2C*, an important transcription factor regulating early neuronal differentiation and cortical lamination (Li et al., 2008). Interestingly, *EPHA3* was also identified as a DEX gene in trisomic excitatory neurons in a recent study (Palmer et al., 2021). It is noteworthy that Palmer et al. performed scRNA-seq on postmortem brain tissue from individuals with DS, which is vastly different from the *in vitro* samples we used in the current study. However, despite the biological differences, both studies identified *EPHA3* as a downstream factor of HSA21 trisomy, suggesting that cell-cell signaling, and neuronal motility may be a common aspect of DS pathology. This concordance, as well as the many other DEX genes shared between the two studies, indicate that the technical and quantitative approaches that the two studies employed were rigorous and replicable, and that CS as a model system for brain development is valid and promising.

By focusing our study on isogenic cell lines, we eliminated confounding biological factors such as sex, ethnicity, somatic variability, etc. To demonstrate this unique aspect of our study, we compared the current dataset to three previously published transcriptomics datasets. We first examined DEX genes on HSA21 and found statistically significant overlap between every pair of datasets, including 16 genes that were shared by all datasets. The observation suggests that a largely consistent cohort of HSA21 genes are dysregulated in neocortex or cortical model systems, regardless of age, sex or experimental system. Since dysregulation of HSA21 is the root cause of DS phenotypes, the remarkable overlap of HSA21 DEX genes we observed not only cross-validates the findings of the included studies, but also highlights the 16 shared DEX genes as key candidates for DS in the brain. Conversely, the fact that out of all triplicated HSA21 genes only 16 were present in all three datasets implies that gene dosage effect in trisomy 21 varies between cell types, tissue, and developmental stage.

Comparing non-HSA21 DEX genes from the studies, we once again identified statistically significant overlap between DEX genes from our CS dataset and those from the Klein et al. (2022) (D8), as well as those from Palmer et al. (2021) (all and old fem.). We also found significant overlap between the Palmer et al. (2021) dataset with the Klein et al. (2022) dataset. The only dataset that did not have statistically significant overlap with our current was Olmos-Serrano et al. (2016). Similarly, the overlap between the Klein et al. (2022) dataset with Olmos-Serrano et al. (2016) was also much weaker. Since Olmos-Serrano et al. (2016) was the only dataset generated with microarray technology, the lack of overlap in non-HSA21 DEX genes may be largely due to technical differences between microarray and RNA-seq approaches.

Next, we wanted to understand the impact of biological sex on transcriptional variability in the studies. In the current study, we focused our analysis on isogenic lines of CS derived from a female individual. By doing so, we eliminated the potential influence of sex on the global transcriptome, which allowed us to distill transcriptomic changes specific to the woman the sample was derived from. To illustrate the potential influence of sex on transcriptomics data, we reanalyzed scRNA-seq data of only the female samples from the Palmer et al. (2021) (age range of 39–60) and compared it to the entire Palmer et al. (2021) dataset with samples from both sexes. Surprisingly, we observed that while 5,429 genes were DEX in the Palmer et al. (2021) (all), 3,280 genes were DEX in Palmer et al. (2021) (old fem.), and only 2,143 DEX genes were shared between the two. These findings support the effect biological sex and age has on the transcriptome of individuals with DS, demonstrating that these variables should be controlled in future studies, regardless of whether they are focused on gene expression or on cellular/anatomical datasets.

Another aspect of experimental design that may influence the power of the analyses is the variability of individual genetic background of the subjects. We sought to minimize the effect of individual genetic background by studying isogenic cell lines derived from the same individual. We demonstrated the advantage of this approach by comparing DEX genes we identified in the current study to those identified in Klein et al. (2022), in which the same isogenic lines were used. While the cell lines were exposed to completely different culturing and differentiation conditions and were analyzed by different technologies, we observed the most significant overlap of DEX genes between our study and Klein et al. (2022). Furthermore, even though we observed nearly perfect overlap of DEX genes on HSA21, the overlap for non-HSA21 genes (while still significant), was less substantial. This indicates that features of dysregulation across the genome are affected by background genetics and environmental conditions. We must acknowledge two limitations of the current study. The first one is the usage of one isogenic pair, containing one euploid and one trisomic line, and the second is the usage of CS derived from the same differentiation experiment for our scRNA-seq experiment. Both confinements limit our ability to draw broader conclusions. Still, an attention to genetic predisposition and addressing the individualized aspects of DS in a “personalized” manner and inclusion of more isogenic lines should be an important consideration for future studies.

Despite the differences between datasets, we identified ten genes on non-HSA21 chromosomes that were dysregulated all studies: *GRIK3*, *NFE2L2*, *SEMA5B*, *POU6F2*, *HECW1*, *BDNF*, *ARL4D*, *JUND*, *COMT*, and *PRKX*. As these ten genes are consistently dysregulated across age, sex, sample type, and sequencing technology, they may strongly impact the consistent neurodevelopmental phenotypes in DS leading to the intellectual disability.

A few of these genes have been reported before in conjunction with DS. Increased levels of *COMT*, encoding catechol-*O*-methyltransferase, has been reported in erythrocytes in individuals with DS (Gustavson et al., 1982). Intriguingly, this enzyme is active in the prefrontal cortex and is responsible for metabolizing catecholamine neurotransmitters. Mutations in *COMT* have previously been associated with executive dysfunction and schizophrenia (Bearden et al., 2004; Baker et al., 2005; Craddock et al., 2006). Increased levels of COMT activity in DS may disrupt homeostatic levels of important neurotransmitters, impairing neural connectivity. NFE2L2 has previously been implicated in the development of Alzheimer’s pathology in DS (Sharma et al., 2020; Lanzillotta et al., 2021) and decreased mRNA and protein levels of BDNF have long been linked to deficits in learning and memory in mouse models of DS (Bimonte-Nelson et al., 2003; Bianchi et al., 2010; Parrini et al., 2017). Decreased levels of JUND have been reported in samples from brains of individuals with DS (Labudova et al., 1998). As JUND has been shown to protect cells from apoptosis (Weitzman et al., 2000), decrease in its expression is in line with the increase in apoptosis we observed in our trisomic CS.

The other six DEX genes in common from all four datasets have not been previously associated with DS. However, most of them have been shown to play important roles in neurodevelopment and their dysregulation in trisomy 21 may be a common mechanism contributing to the neurological changes and ID in all individuals with DS. *POU6F2* encodes a transcription factor important in neural subtype determination (Fiorino et al., 2016; Ainsworth et al., 2018). *GRIK3* encodes a glutamate receptor subunit. Gain of function mutations of *GRIK3* have previously been associated with ID and neurodevelopmental deficits (Shibata et al., 2006). *SEMA5B* is important for axon guidance and cell migration, which we also identified as key dysregulated features in ExN from our trisomic CS (Yazdani and Terman, 2006). The recurrent dysregulation of the ten DEX genes across multiple datasets suggest their function and dysfunction may be key to understanding common aspects of the neurodevelopmental deficits across all individuals with the neurodevelopmental deficits characteristic of DS.

Altogether our study demonstrates the power of deeply analyzing genetically defined isogenic CS in a personalized manner, in conjunction with broad examination of transcriptional dysregulation, in the context of DS. As transcriptional dysregulation in neurodevelopmental diseases such as DS varies not only between individuals but also between tissue and cell types, it is also important to examine transcriptomic changes at the cellular level to gain functionally relevant and clinically actionable insights. At the same time, the variability across technological and experimental conditions and its influence on the interpretation of results should not be underestimated. Our study identifies multiple genes of interest consistent across datasets. Future studies will be necessary to confirm and elucidate their role in neurodevelopmental phenotypes in DS.

## Data availability statement

The scRNA-seq data for this study can be found in the Sequence Read Archive (SRA) with access number: PRJNA828127. The Palmer et al. (2021) dataset was downloaded from European Genome-Phenome Archive (EGAD00001008287) with approval.

## Author contributions

EZ and TFH conceived the idea. JAK, EZ, NC, and RK performed all the tissue culture experiments. ZL, SR, and YP assisted with data analysis and performed the bioinformatics analysis. ZL, JAK, EZ, and TFH wrote the manuscript. All authors contributed to the article and approved the submitted version.

## Footnotes

1
https://www.bumc.bu.edu/anatneuro/ella-zeldich-lab/
